# Polyunsaturated Fatty Acid Levels in Maternal Erythrocytes of Japanese Women during Pregnancy and after Childbirth

**DOI:** 10.3390/nu9030245

**Published:** 2017-03-07

**Authors:** Terue Kawabata, Yasuo Kagawa, Fumiko Kimura, Teruo Miyazawa, Shoji Saito, Takahiro Arima, Kunihiko Nakai, Nobuo Yaegashi

**Affiliations:** 1 Faculty of Nutrition, Kagawa Nutrition University, 3-9-21 Chiyoda, Sakado, Saitama 350-0288, Japan; kagawa@eiyo.ac.jp; 2 Faculty of Comprehensive Human Sciences, Shokei Gakuin University, 4-10-1 Yurigaoka, Natori, Miyagi 981-1295, Japan; f_kimura@shokei.ac.jp; 3 Food and Biodynamic Chemistry Laboratory, Graduate School of Agricultural Sciences, Tohoku University, 468-1 aza-Aoba, Aramaki, Aoba-ku, Sendai, Miyagi 980-0845, Japan; miyazawa@m.tohoku.ac.jp; 4 New Industry Creation Hatchery Center (NICHe), Tohoku University, 6-6-10 aza-Aoba, Aramaki, Aoba-ku, Sendai, Miyagi 980-8579, Japan; 5 Department of Gynecology and Obstetrics, Tohoku University Graduate School of Medicine, 2-1 Seiryo-machi, Aoba-ku, Sendai, Miyagi 980-8575, Japan; shojisaiko5324@yahoo.co.jp (S.S.); yaegashi@med.tohoku.ac.jp (N.Y.); 6 Department of Obstetrics and Gynecology, Yamagata Prefectural Central Hospital, 1800 Ooaza-Aoyagi, Yamagata-shi, Yamagata 990-2292, Japan; 7 Department of Informative Genetics, Tohoku University Graduate School of Medicine, 2-1 Seiryo-machi, Aoba-ku, Sendai, Miyagi 980-8575, Japan; tarima@med.tohoku.ac.jp; 8 Department of Development and Environmental Medicine, Tohoku University Graduate School of Medicine, 2-1 Seiryo-machi, Aoba-ku, Sendai, Miyagi 980-8575, Japan; nakaik@med.tohoku.ac.jp

**Keywords:** pregnancy, polyunsaturated fatty acid (PUFA), docosahexaenoic acid (DHA), arachidonic acid (ARA), Japanese

## Abstract

Background: The transport of polyunsaturated fatty acids (PUFAs), such as arachidonic acid (ARA, 20:4n-6) and docosahexaenoic acid (DHA, 22:6n-3), to the fetus from maternal stores increases depending on the fetal requirements for PUFA during the last trimester of pregnancy. Therefore, maternal blood PUFA changes physiologically with gestational age. However, the changes in PUFA levels in maternal blood erythrocytes during pregnancy and after childbirth have not been fully investigated in a fish-eating population. Objective: To examine the changes of ARA and DHA levels in maternal erythrocytes with the progress of pregnancy and the relationship between maternal and umbilical cord erythrocyte PUFA levels in pregnant Japanese women who habitually eat fish and shellfish. Design: This study was performed as a part of the adjunct study of the Japan Environment and Children’s Study (JECS). The participants were 74 pregnant women. The maternal blood samples were collected at 27, 30, and 36 weeks of pregnancy, and 2 days and 1 month after delivery, and umbilical cord blood was collected at delivery. The fatty acid levels of erythrocytes in these blood samples were determined. Results: ARA and DHA levels in maternal erythrocytes tended to decrease with the progress of pregnancy. While the DHA level decreased further after delivery, the ARA level returned to the value at 27 weeks of pregnancy within 1 month after delivery. The n-3 and n-6 PUFA levels in maternal erythrocytes at 27, 30, and 36 weeks of pregnancy were significantly positively correlated with the corresponding fatty acid levels in umbilical cord erythrocytes. Conclusion: The present findings showed a significant change in erythrocyte PUFA levels during pregnancy and after childbirth in a fish-eating population. The PUFA levels of maternal blood after the second trimester may be a reliable marker for predicting PUFA levels in infants’ circulating blood.

## 1. Introduction

Polyunsaturated fatty acids (PUFAs), such as arachidonic acid (ARA, 20:4n-6) and docosahexaenoic acid (DHA, 22:6n-3), are essential structural components of cell membranes of the brain and retina [1]. DHA is especially important for normal nervous and visual function, and it is known that DHA is accumulated rapidly in the fetal retina and brain during the last trimester of pregnancy [2,3]. The results of previous studies have suggested positive associations between fetal blood or tissue DHA contents, which could be supplied from maternal stores during pregnancy, and neurological and motor functions in later life [4,5]. Therefore, it is very important that maternal stores be large enough through maternal dietary DHA intake during pregnancy.

Japanese people habitually eat fish and shellfish that are rich in n-3 PUFAs including DHA [6,7]. According to the National Health and Nutritional Survey of Japan [6], the mean intake of fish and shellfish by adults aged 20 years and over was 81.7 g/day for men and 67.8 g/day for women. In the dietary records, together with photographic records of 28 consecutive days, we have previously demonstrated that the mean fish intake of young women of about 20 years of age was 42.8 g/day, and the DHA composition of erythrocyte phospholipids measured simultaneously was 6.1% [7]. In addition, it has been reported that the erythrocyte DHA composition in Japanese people was 7.0% in less than 35-year-old males [8], 6.4% in 20 to 60-year-old women [9], and 7.9% in middle-aged men and women [10]. It has been widely shown that there is an association between dietary n-3 PUFA intake and n-3 PUFA composition in blood [11,12,13,14]. Lands [15] showed a positive correlation of the percentage of n-3 fatty acids in blood 20- and 22-carbon PUFAs and the daily menu balance values determined by the balance of n-6 and n-3 fatty acids (en%) in food; the daily menu balance value of traditional Japanese foods was highly positioned, next to that of Greenland. Thus, it is clear that Japanese people sustain high blood levels of DHA due to habitual intake of fish and shellfish.

Several previous studies indicated that maternal PUFA levels in blood change uniquely during pregnancy and after delivery [16,17,18,19,20,21,22,23,24,25,26,27]. A typical previous study showed that maternal plasma essential fatty acid (EFA) and PUFA levels decreased continuously throughout pregnancy [16]. This was considered to be due to the rapid increase of EFA and PUFA transfer to the fetus from the maternal stores to satisfy fetal requirements for EFAs and PUFAs during the last trimester of pregnancy [16,19,27]. Since maternal blood fatty acid levels change physiologically with gestational age, it is necessary to know how maternal blood fatty acid levels during pregnancy affect fatty acid levels in newborn circulating blood at delivery. Most previous publications have investigated DHA levels in maternal erythrocytes, with results generally of 2% to 4% [18,19,20,22,23,24,25,26]. On the other hand, Markhus et al. [27] reported that the PUFA levels in blood in women during gestation and postpartum were 6.0%–7.2% in erythrocyte DHA, namely the values of this DHA were almost the same as in Japanese people in general. In this study, the maternal DHA status decreased from the last trimester of pregnancy to the time of lactation, and then increased from six months after delivery to one year. Although there are a few reports of study populations whose DHA status was comparatively high [27], the longitudinal changes in PUFA levels throughout pregnancy and delivery should be confirmed in a population that consumes fish regularly.

The main purpose of this study was to examine the changes of ARA and DHA levels in maternal erythrocytes with the progress of pregnancy in pregnant Japanese women. In addition, we have been following a birth cohort study to examine the beneficial effects of maternal DHA on child development as an Adjunct study of a national birth cohort study, the Japan Environment and Children’s Study (JECS) [28]. In these birth cohort situations, cord blood PUFA levels might be important for childhood growth and development. However, blood samples are sometimes lacking for various reasons during delivery. In this context, a method to estimate PUFA levels in umbilical cord erythrocytes based on PUFA levels in maternal blood is needed. Therefore, the second purpose of this study was to determine whether the PUFA levels of maternal blood during pregnancy can predict the PUFA levels in umbilical cord blood. 

## 2. Materials and Methods

### 2.1. Study Design

The present study was performed as a part of the Adjunct study outlined in the JECS protocol paper [28]. The Adjunct study protocol was approved by the Ministry of the Environment [29]. The pregnant women (first trimester to 12 weeks of gestation) who visited antenatal hospitals in the research area of Miyagi Prefecture were initially recruited to JECS, and the JECS participants in the coastal area (3029 pregnant women) were further recruited into the Adjunct study. Finally, of 1878 participants in the Adjunct study, 74 healthy pregnant women who visited an antenatal clinic from March 2012 to December 2013 were included in this study. Their relevant clinical information is summarized in Table 1. The study protocol was approved by two ethics committees: the Ethics Committee of the Tohoku University Graduate School of Medicine (No. 2010-322/25 October 2010，No. 2010-421/17 December 2010 and No. 2012-1-154/13 July 2012) and the Medical Ethics Committee of Kagawa Nutrition University (No. 187G/18 January 2012). All information regarding the study was given to each participant who voluntarily agreed to participate and provided written, informed consent.

### 2.2. Blood Sampling

The pregnant participants were regularly examined in the antenatal clinic, and blood samples were drawn for routine gynecological check-ups at 27, 30, and 36 weeks of pregnancy, and 2 days and 1 month after delivery. When residual blood was available, it was used for this study. Cord blood was also collected from the umbilical cord vein immediately after delivery. All blood samples were collected into ethylenediaminetetraacetic acid (EDTA)-2Na-containing tubes. These samples were then taken to the analysis institution (Kagawa Nutrition University) at 4 °C within 4 days, and fatty acid levels were analyzed immediately on arrival. 

### 2.3. Fatty Acid Analysis

Whole blood was centrifuged at 1600× *g* for 10 min to separate plasma and erythrocytes. The erythrocytes were washed twice with physiological saline, and the buffy coat was removed. According to the method of Rose and Oklander [30], the erythrocyte lipid was extracted using isopropanol/chloroform. Briefly, distilled water with 0.1 mM EDTA-2Na was added to the erythrocytes and mixed. After 15 min, isopropanol was added slowly with mixing and allowed to stand for 1 h. Then, chloroform was added and mixed. After another hour, the lipid was transferred into chloroform-methanol 2:1 (*v*/*v*) and washed with distilled water with 0.9% KCl. The tube was centrifuged at 1600× *g* for 5 min, and the lipid extract was obtained in the lower phase. The individual lipid extracts were methylated using methanol hydrochloride, and then gas–liquid chromatography analysis of the fatty acid methyl ester levels was conducted, as described previously [31].

### 2.4. Statistical Analysis

The fatty acid levels of erythrocytes in maternal and umbilical cord blood are expressed as percentages of the total fatty acids. Because the data were not normally distributed, data are presented as medians (interquartile range, 25th–75th percentiles). Comparisons between maternal and umbilical cord erythrocyte fatty acids were conducted using the Kruskal–Wallis test. To examine the longitudinal changes in maternal status, Friedman’s analysis was conducted, followed by a post hoc analysis to identify significant differences between blood sampling periods using the Wilcoxon rank-sum test with Bonferroni correction. The relationships between maternal and umbilical cord erythrocyte fatty acids were examined by Spearman’s rank correlation. The correlation coefficients were calculated only if the medians of the fatty acid levels were more than 1% in both maternal and umbilical cord erythrocytes, except for eicosapentaenoic acid (EPA, 20:5n-3).

Significance was defined as *p* < 0.05. All statistical analyses were carried out using the JMP software package, version 10.0 (SAS Institute Inc., Cary, NC, USA).

## 3. Results

**Figure 1:**
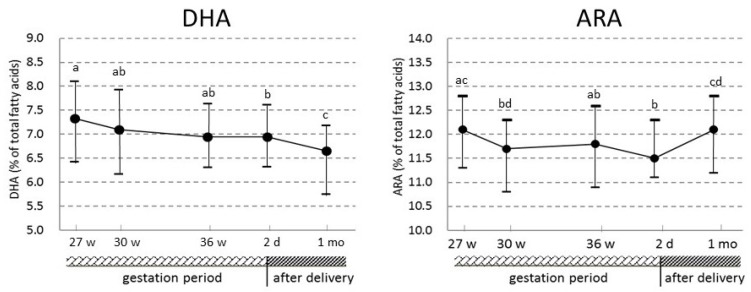
The changes in docosahexaenoic acid (DHA) and arachidonic acid (ARA) levels in maternal erythrocytes during gestation and after delivery. Values are expressed as medians (inter-quartile range) (*n* = 74). The longitudinal changes in maternal status were examined by Friedman’s test (*p* values), and a post hoc analysis was conducted to identify significant differences between each blood sampling period using the Wilcoxon rank-sum test with Bonferroni correction. Median values indicated by dissimilar letters are significantly different (*p* < 0.05).

Fatty acid levels in umbilical cord and maternal erythrocytes and the correlation coefficients for each corresponding erythrocyte fatty acid between umbilical cord and maternal erythrocytes at 27, 30, and 36 weeks of pregnancy and 2 days and 1 month after delivery are shown in Table 2. Most fatty acid levels in maternal erythrocytes changed significantly during gestation and after delivery. The changes in ARA and DHA levels, which are essential nutrients for the fetus and infants, are also shown in Figure 1. ARA and DHA levels in maternal erythrocytes tended to decrease with the progress of pregnancy, and the levels at 2 days after delivery were significantly decreased in comparison with the levels at 27 weeks of pregnancy. After delivery, the DHA level was also significantly decreased compared with that during pregnancy. However, the ARA level in maternal erythrocytes at 1 month after delivery was significantly higher than at 36 weeks of pregnancy and 2 days after delivery, and it returned to the value at 27 weeks of pregnancy within four weeks after delivery. The ARA and DHA levels in maternal erythrocytes at 1 month after delivery were not significantly different among breast-, bottle- and mixed-feeding (median values: 12.28, 11.37, and 11.91 for ARA, 7.12, 6.41, and 6.46 for DHA, respectively). 

Levels of all fatty acids in the n-3 PUFA and n-6 PUFA groups and total n-3 PUFA and n-6 PUFA in maternal erythrocytes at 27, 30, and 36 weeks of pregnancy and 2 days and 1 month after delivery were significantly positively correlated with those of the corresponding fatty acids in umbilical cord erythrocytes (Table 2). Except for the ARA level at 1 month after delivery, the strengths of the correlations were almost the same regardless of the time at which the maternal blood samples were drawn. 

## 4. Discussion

The present study showed that (1) DHA and ARA levels in maternal erythrocytes decreased with the progress of pregnancy, and then DHA decreased further after delivery, while ARA returned to the value at 27 weeks of pregnancy within four weeks after delivery; (2) for fatty acids in the n-3 and n-6 PUFA groups, the levels in maternal erythrocytes at 27, 30, and 36 weeks of pregnancy were significantly positively correlated with the corresponding fatty acid levels in umbilical cord erythrocytes, and the strengths of these correlations were almost the same regardless of the period that maternal blood was drawn.

DHA and ARA levels in maternal erythrocytes decreased significantly in the third trimester. In the previous reports examining changes in ARA levels in maternal erythrocytes during pregnancy, it was shown that ARA levels decreased from the second trimester to childbirth [16,19,20,21,22,23]. Similar results have also been reported for DHA [16,19,32]. Generally, the decreases in the relative levels of ARA and DHA in maternal erythrocytes in the third trimester are thought to be due to accelerated transport of these fatty acids to the fetus [33]. On the other hand, several previous studies showed opposite results, with DHA levels in maternal blood being unchanged or increasing [18,20,21,22,23,24,25]. Under conditions of persistently low maternal DHA status, the DHA levels at delivery of the mother who bore premature infants and full-term-birth infants were almost the same [34]. This means that DHA in maternal blood did not decrease in the last part of the third trimester of pregnancy, and it was thought that there was no DHA transfer from the maternal stores to the fetus due to DHA deficiency. In another typical study, pregnant women of South India, who mostly do not consume fish, had lower DHA levels (approximately 2%) in their erythrocytes, but their erythrocyte DHA concentrations did not decline from the second to third trimesters [22]. It was suggested that the conversion of α-linolenic acid to DHA was increased during pregnancy as an adaptation to the very low ingestion of DHA in that population. The median DHA in maternal erythrocytes in the present study was high (approximately 7%). Therefore, DHA transfer to the fetus from the maternal stores must have occurred continuously just before childbirth. Further, it is hard to think that the conversion of α-linolenic acid to DHA was markedly increased as a compensatory action with insufficient maternal DHA stores. It was considered that the DHA decrease during the last trimester of pregnancy in these research subjects directly reflected DHA transfer to the fetus from the mother.

Moreover, the present study indicated that the DHA level in maternal erythrocytes at 1 month after delivery was significantly decreased compared to that during pregnancy, and the ARA level returned to the previous value observed at 27 weeks of pregnancy within four weeks after delivery. Similar changes were reported in several previous studies [23,24,25,26]. The decreased DHA level might be caused by increased demand accompanying maternal milk secretion [26,27,35]. In the meantime, with respect to the decrease in the absolute value of plasma DHA from delivery to 3 months’ postpartum, Stark et al. [25] suggested that there was no difference between breastfeeding and non-breastfeeding women. In the present subjects, no difference in DHA levels was also seen among breast-, bottle-, and mixed-feeding subjects. Even if a fish oil supplement were given to mothers, and their erythrocyte DHA levels were maintained at a high level during pregnancy, there have been reports of decreased DHA levels after childbirth [21]. These changes in DHA levels might be hormonally mediated [25,36]. Previous studies have shown that it was possible to increase the activity of the desaturation and chain-elongation pathway by estrogen [37,38]. It has also been reported that the activity of retroconversion of DHA to EPA differs between menopausal women on estrogen-replacement therapy and menopausal women not on estrogen-replacement therapy [39]. Thus, a hormone such as estrogen may have a large effect on fatty acid metabolism. Meanwhile, another report showed that the DHA status might be highly maintained during pregnancy by the conversion of EPA to DHA, and a decrease of this conversion occurs after delivery, causing the DHA in blood to also decrease after childbirth [25]. In any case, at this stage, the reasons for the decreased DHA after delivery observed in the present study are not clear. In the present subjects, exclusive bottle-feeding was observed in only four lactating women; therefore, further investigations with an increased number of participants are needed.

In the present study, levels of all fatty acids in the n-3 PUFA and n-6 PUFA groups in maternal and umbilical cord blood erythrocytes were positively correlated. A correlation between PUFA levels in the maternal blood and in the umbilical cord blood has been demonstrated [18,20,40,41,42,43]. Regardless of the timing of maternal blood sampling, the PUFA level in maternal erythrocytes could predict the umbilical cord blood erythrocyte PUFA level from the viewpoint of individual differences within a population group. However, when attempting to predict erythrocyte PUFA levels in umbilical cord blood, it becomes an indispensable condition that the dietary habits from the period of drawing blood to the time of childbirth remain essentially the same, because it is known that EPA and DHA levels in blood tend to be affected by dietary fish intake [11,13,14]. When we predict PUFA levels in infants using maternal blood sampled during pregnancy, but not at the time of delivery, previous studies reported that PUFA levels in erythrocytes were better than plasma PUFA levels [20]. Indeed, erythrocytes were used in the present study. The present finding is important for epidemiological studies because maternal blood at delivery and umbilical cord blood can be lost due to various obstetric situations.

## 5. Conclusions

In conclusion, ARA and DHA levels in maternal blood decreased from the end of the second trimester to delivery, and then DHA decreased further after delivery. Further, the strengths of the correlations with the PUFA levels in maternal blood at every blood sampling period during pregnancy and in umbilical cord blood did not differ. The present findings in pregnant women showed that PUFA levels in maternal erythrocytes changed with the progress of pregnancy, but the erythrocyte PUFA level in maternal circulating blood from the end of the second trimester to delivery could be a reliable marker to predict fatty acid levels in newborn infants’ circulating blood.

## Figures and Tables

**Table 1:** Characteristics of the study population (*n* = 74).

	*n* (%)	Median	(Inter-Quartile Range)
Maternal age (years)		29.6	(25.9–33.9)
Prepregnancy height (cm)		158	(152.8–162.1)
Prepregnancy weight (kg)		53.1	(48.5–60.7)
Prepregnancy body mass index (kg/cm^2^)		21.8	(19.9–24.4)
Body weight gain (kg)		11.6	(8.9–14.2)
Gestational period (day)		278	(274–285)
Birth height (cm)		49.5	(48–50.5)
Birth weight (g)		3170	(3004–3494)
Head circumference (cm)		34	(33–35)
Chest circumference (cm)		32	(31–33)
Sex, female	42 (56.8)		
male	32 (43.2)		
Nursing, breast-feeding	13 (17.6)		
bottle-feeding	4 (5.4)		
mixed-feeding	57 (77.0)		

**Table 2:** Fatty acid levels (% of total fatty acids) in umbilical cord and maternal erythrocytes and the correlation coefficients for each corresponding erythrocyte fatty acid between umbilical cord and maternal erythrocytes at 27, 30, and 36 weeks of pregnancy and 2 days and 1 month after delivery.

	Umbilical Cord Erythrocytes (*n* = 74)	Maternal Erythrocytes															
		27 Weeks of Gestation (*n* = 74)			30 Weeks of Gestation (*n* = 74)			36 Weeks of Gestation (*n* = 74)			2 Days after Delivery (*n* = 74)			1 Month after Delivery (*n* = 74)			*p* ^2^
		Median (Inter, Quartile Range)	Correlation Umbilical Cord vs. Maternal		Median (Inter, Quartile Range)	Correlation Umbilical Cord vs. Maternal		Median (Inter, Quartile Range)	Correlation Umbilical Cord vs. Maternal		Median (Inter, Quartile Range)	Correlation Umbilical Cord vs. Maternal		Median (Inter, Quartile Range)	Correlation Umbilical Cord vs. Maternal		
			*r*	*p* ^1^		*r*	*p* ^1^		*r*	*p* ^1^		*r*	*p* ^1^		*r*	*p* ^1^	
SFA	51.5 (50.3–52.6)	46.7 (46.2–47.6)	0.070	0.553	46.5 (45.7–47.5)	0.071	0.548	46.9 (46.2–47.6)	0.183	0.118	47.1 (46.1–47.9)	0.277	0.017	47.0 (46.1–47.9)	-0.004	0.971	0..091
16:0	26.9 (26.3–27.6)	24.6 ^a^ (24.2–25.2)	-0.128	0.279	24.9 ^ab^ (24.3–25.6)	0.107	0.365	25.0 ^b^ (24.4–25.6)	0.202	0.085	25.4 ^c^ (24.7–26.0)	0.152	0.196	24.5 ^a^ (24.1–25.1)	-0.076	0.520	<0.001
18:0	17.9 (17.5–19.4)	16.0 ^ac^ (15.4–16.8)	0.085	0.473	15.6 ^ab^ (15.1–16.5)	-0.109	0.355	15.7 ^b^ (15.0–16.3)	-0.082	0.490	15.5 ^b^ (14.9–16.2)	0.186	0.112	16.4 ^c^ (15.9–17.3)	-0.027	0.818	<0.001
22:0	1.05 (0.99–1.17)	1.21 ^a^ (1.10–1.32)	0.127	0.281	1.21 ^a^ (1.10–1.33)	-0.038	0.746	1.29 ^b^ (1.16–1.41)	0.215	0.066	1.17 ^a^ (1.10–1.32)	0.304	0.009	1.20 ^a^ (1.12–1.30)	0.089	0.450	<0.001
24:0	3.73 (3.43–4.08)	3.37 ^a^ (3.20–3.65)	0.096	0.417	3.44 ^a^ (3.25–3.66)	0.028	0.814	3.56 ^b^ (3.31–3.80)	0.102	0.388	3.44 ^a^ (3.21–3.59)	0.250	0.032	3.31 ^a^ (3.13–3.53)	0.092	0.438	<0.001
MUFA	15.1 (14.5–15.9)	18.4 ^a^ (17.7–19.1)	0.037	0.756	18.9 ^bc^ (18.0–19.6)	0.043	0.719	19.0 ^c^ (18.5–19.6)	-0.027	0.820	19.0 ^bc^ (18.3–19.6)	0.068	0.568	18.6 ^ab^ (17.9–19.1)	0.010	0.930	<0.001
18:1n-9	9.90 (9.40–10.4)	12.6 ^a^ (12.0–13.1)	0.168	0.153	13.0 ^b^ (12.3–13.5)	0.170	0.149	13.1 ^b^ (12.5–13.6)	0.052	0.661	13.1 ^b^ (12.5–13.6)	0.112	0.343	12.8 ^b^ (12.4–13.5)	0.065	0.583	<0.001
18:1n-7	1.59 (1.44–1.72)	1.08 ^ab^ (1.02–1.15)	0.241	0.039	1.10 ^a^ (1.04–1.18)	0.337	0.003	1.07 ^b^ (1.00–1.15)	0.294	0.011	1.04 ^b^ (0.99–1.12)	0.331	0.004	1.11 ^a^ (1.03–1.17)	0.119	0.311	<0.001
24:1n-9	2.99 (2.74–3.34)	4.10 ^ac^ (3.82–4.36)	0.067	0.570	4.18 ^ab^ (3.76–4.50)	0.101	0.390	4.25 ^b^ (4.04–4.49)	0.089	0.450	4.13 ^ab^ (3.90–4.40)	0.155	0.187	3.97 ^c^ (3.68–4.20)	0.066	0.577	<0.001
PUFA	33.3 (32.5–34.4)	34.7 ^a^ (34.0–35.4)	0.146	0.216	34.6 ^ab^ (33.4–35.6)	0.304	0.009	34.1 ^b^ (33.4–35.0)	0.314	0.006	33.8 ^b^ (33.1–35.1)	0.217	0.064	34.3 ^ab^ (33.7–35.3)	0.202	0.085	<0.001
n-3 PUFA	7.85 (6.92–8.87)	9.98 ^a^ (8.84–11.2)	0.573	<0.001	9.54 ^ab^ (8.38–10.7)	0.463	<0.001	9.37 ^bc^ (8.67–10.6)	0.550	<0.001	9.46 ^bc^ (8.83–10.6)	0.543	<0.001	9.44 ^c^ (8.35–10.6)	0.639	<0.001	<0.001
20:5n-3	0.25 (0.18–0.33)	0.65 ^a^ (0.47–0.89)	0.819	<0.001	0.64 ^a^ (0.45–0.89)	0.791	<0.001	0.55 ^b^ (0.46–0.80)	0.811	<0.001	0.61 ^ab^ (0.50–0.81)	0.858	<0.001	0.87 ^c^ (0.62–1.17)	0.630	<0.001	<0.001
22:5n-3	0.65 (0.54–0.85)	1.85 ^a^ (1.66–2.05)	0.544	<0.001	1.74 ^ab^ (1.63–1.95)	0.441	<0.001	1.76 ^ab^ (1.62–1.94)	0.529	<0.001	1.78 ^b^ (1.62–1.92)	0.520	<0.001	1.79 ^ab^ (1.63–2.06)	0.564	<0.001	<0.001
22:6n-3	6.92 (6.14–7.45)	7.33 ^a^ (6.43–8.11)	0.440	<0.001	7.10 ^ab^ (6.17–7.93)	0.370	<0.001	6.95 ^ab^ (6.31–7.64)	0.410	<0.001	6.95 ^b^ (6.32–7.62)	0.432	<0.001	6.66 ^c^ (5.75–7.19)	0.539	<0.001	<0.001
n-6 PUFA	25.2 (24.2–26.3)	24.6 ^ab^ (23.5–25.8)	0.362	0.002	24.6 ^ab^ (23.3–25.7)	0.443	<0.001	24.5 ^ab^ (23.4–25.3)	0.485	<0.001	24.4 ^a^ (23.2–25.3)	0.516	<0.001	24.6 ^b^ (23.2–25.6)	0.380	0.001	0.002
18:2n-6	4.03 (3.56–4.34)	8.88 ^a^ (8.39–9.41)	0.444	<0.001	9.08 ^bc^ (8.64–9.90)	0.440	<0.001	8.95 ^ab^ (8.39–9.52)	0.478	<0.001	9.08 ^b^ (8.51–9.73)	0.615	<0.001	9.25 ^c^ (8.58–9.96)	0.420	<0.001	<0.001
20:3n-6	2.38 (2.05–2.69)	1.36 ^ac^ (1.22–1.50)	0.392	0.001	1.38 ^ab^ (1.23–1.53)	0.437	<0.001	1.34 ^c^ (1.19–1.52)	0.509	<0.001	1.35 ^c^ (1.19–1.49)	0.456	<0.001	1.17 ^d^ (1.02–1.36)	0.404	<0.001	<0.001
20:4n-6	15.4 (14.7–16.4)	12.1 ^ac^ (11.3–12.8)	0.349	0.002	11.7 ^bd^ (10.8–12.3)	0.414	<0.001	11.8 ^ab^ (10.9–12.6)	0.361	0.002	11.5 ^b^ (11.1–12.3)	0.379	0.001	12.1 ^cd^ (11.2–12.8)	0.247	0.034	<0.001
22:4n-6	2.93 (2.61–3.30)	2.06 ^a^ (1.77–2.33)	0.471	<0.001	1.99 ^ab^ (1.71–2.25)	0.561	<0.001	1.98 ^ab^ (1.67–2.30)	0.539	<0.001	1.94 ^b^ (1.65–2.26)	0.588	<0.001	1.83 ^c^ (1.60–2.09)	0.566	<0.001	<0.001

SFA, saturated fatty acids; MUFA, monounsaturated fatty acids; PUFA, polyunsaturated fatty acids. ^1^ Spearman’s correlation coefficients for each corresponding erythrocyte fatty acid between umbilical cord and maternal erythrocytes at 27, 30, or 36 weeks of pregnancy or 2 days or 1 month after delivery. ^2^ The longitudinal changes in maternal status were examined by Friedman’s test (*p* values), and a post hoc analysis was conducted to identify significant differences between each blood sampling period using the Wilcoxon rank-sum test with Bonferroni correction. ^a,b,c,d^ Median values within a row with unlike superscript letters are significantly different (*p* < 0.05).
